# Improving ruminal digestibility of various wheat straw types by white‐rot fungi

**DOI:** 10.1002/jsfa.9320

**Published:** 2018-10-16

**Authors:** Nazri Nayan, Gijs van Erven, Mirjam A Kabel, Anton SM Sonnenberg, Wouter H Hendriks, John W Cone

**Affiliations:** ^1^ Animal Nutrition Group Wageningen University & Research Wageningen The Netherlands; ^2^ Laboratory of Food Chemistry Wageningen University & Research Wageningen The Netherlands; ^3^ Plant Breeding Wageningen University & Research Wageningen The Netherlands

**Keywords:** white‐rot fungi, wheat straw cultivar, wheat straw maturity, *in vitro* gas production, ruminant feed, lignocellulosic biomass

## Abstract

**BACKGROUND:**

This study investigated the ruminal degradability of various wheat straw types by the white‐rot fungi *Ceriporiopsis subvermispora* (CS) and *Lentinula edodes* (LE). Different cultivars (CV) of wheat straw at different maturity stages (MS) were treated with the fungi for 7 weeks and assessed for chemical composition and *in vitro* gas production (IVGP).

**RESULTS:**

Both fungi showed a more pronounced degradation of lignin on a more mature straw (MS3; 89.0%) in comparison with the straw harvested at an earlier stage (MS1; 70.7%). Quantitative pyrolysis coupled to gas chromatography and mass spectrometry, using ^13^C lignin as an internal standard ^13^C‐IS Py‐GC/MS revealed that lignin in more mature straw was degraded and modified to a greater extent. In contrast, cellulose was less degraded in MS3, as compared to MS1 (8.3% versus 14.6%). There was no effect of different MS on the IVGP of the fungus‐treated straws. Among the different straw cultivars, the extent of lignin degradation varied greatly (47% to 93.5%). This may explain the significant (*P* < 0.001) effect of cultivar on the IVGP of the fungal‐treated straws. Regardless of the factors tested, both fungi were very capable of improving the IVGP of all straw types by 15.3% to 47.6%, (as compared to untreated straw), with CS performing better than LE – on different MS (33.6% versus 20.4%) and CVs (43.2% versus 29.1%).

**CONCLUSION:**

The extent of lignin degradation caused by fungal treatment was more pronounced on the more mature and lignified straw, while variable results were obtained with different cultivars. Both fungi were capable of improving the IVGP of various straw types. © 2018 The Authors. *Journal of the Science of Food and Agriculture* published by John Wiley & Sons Ltd on behalf of Society of Chemical Industry.

## INTRODUCTION

The valorization of lignocellulosic biomass, such as wheat straw, as an alternative feed ingredient is important for sustainable animal production. To unlock the energy potential of lignocellulose, various physicochemical methods have been used, such as hydrothermal treatment, ammonia fiber expansion, acids, and alkaline media.^1^, ^2^, ^3^ These methods are used to increase the enzymatic accessibility of the structural carbohydrates by breaking the lignin barrier and thus improving the degradability of the feedstock by ruminants. Although they are attractive for industrial‐scale applications due to time efficiency, their potential adverse impact on the environment is a major concern.^4^ The use of biological pretreatments, particular fungi, has become a preferred approach.^5^, ^6^, ^7^ Some strains of white‐rot fungi possess unique capabilities for degrading highly recalcitrant lignocellulosic biomass, whereby lignin is being selectively degraded, increasing the available carbohydrate contents of the biomass.

The effectiveness of a fungal pretreatment can be influenced by many factors, such as fungal strain, substrate, and culture conditions.^6^, ^8^, ^9^ Variability in substrate and culture conditions leads to difficulties in comparing the effectiveness of fungi across different studies.^9^ The quality of the straw, i.e. the ratio of lignin to total carbohydrates, is not only an important factor influencing its digestibility in the rumen^10^ but may also affect the effectiveness of a fungal pretreatment.^9^ Unfortunately, standardizing the straw for an optimal result is difficult and impractical, considering disparities among the straw types and conditions, as well as the differences in post‐harvest residue management.^11^ The present study was conducted to assess the effect of straw quality on lignocellulose degradation by fungi and the subsequent effect on the ruminal degradability of the treated straw.

Straw quality is affected by many factors including maturity stage, cultivar, and other factors related to management and the environment.^12^, ^13^ The maturity of the plant, in particular, correlates with the extent of lignin deposition in the plant tissues.^13^ The total fiber content increases as the plant matures (lower leaf‐to‐stem ratio^14^) and the lignin, cellulose, and hemicellulose composition also changes. As straw consists mainly of fiber (∼85%), changes in the cell‐wall composition with maturity may thus influence the lignocellulose degradation by fungi. Another important factor is the wheat straw genotype. In wheat breeding, nutrient‐use efficiency and disease resistance are among the selection parameters, in contrast to straw quality.^12^, ^15^

Recently, high‐potential strains of the white‐rot fungi species *Ceriporiopsis subvermispora* and *Lentinula edodes* have been selected for the bioprocessing of wheat straw into ruminant feed.^16^ To determine whether these high‐potential strains could perform well across different straw types, two independent trials were carried out in the present study. These trials investigated the effect of two main sources of straw variation, (i) different maturity of the straw around harvest, and (ii) different straw cultivars, on fungal growth and their persistency in improving the *in vitro* degradability of the straw. The lignin degradation characteristics of wheat straw with different maturities were assessed using pyrolysis coupled to gas chromatography with mass spectrometry (Py‐GC/MS).^17^

## MATERIALS AND METHODS

### Effect of different maturity stages

#### *Harvesting wheat straw with different maturities*

Wheat straw (*Triticum aestivum* L.) of the same variety (*Quintus*) was harvested during the summer of 2015 from the experimental field UNIFARM of Wageningen University, Netherlands. Each harvest covered a plot size of 8.5 × 1.5 m. Zadoks *et al*.^18^ codes were used to characterize the maturity stages of the wheat plant. The first harvest was carried out on July 14 (MS1, code 83 – soft dough development, 37.3% dry weight), the second harvest on July 28 (MS2, code 87 – hard dough development, 41.2% dry weight) and the third harvest on August 11 (MS3, code 91 – ripening, 64.6% dry weight). The wheat plants were cut at 10 cm above the ground and the fresh plants were dried on a drying panel for a week (∼60 °C). Prior to chopping, the spike containing the wheat grains was removed, leaving the straw (leaves and stalk) intact. The trimmed straw was chopped into approximately 3 cm pieces.

#### *Fungal pretreatment of the wheat straw*

Cultures of the white‐rot fungi, (from the collection of Plant Breeding, Wageningen University, the Netherlands) *C. subvermispora* (CS; CBS 347.63; origin: USA) and *L. edodes* (LE; sh 03/08; origin: Japan), were maintained on malt agar extract containing 20.0 g L^−1^ of malt extract, 0.5 g L^−1^ of KH_2_PO_4_, 0.5 g L^−1^ of MgSO_4_.7H_2_O, and 0.5 g L^−1^ of Ca(NO_3_)_2_·4H_2_O, (all chemicals were purchased from Sigma‐Aldrich, St. Louis, MO) with pH 5.4 at 24 °C. Once the fungi fully colonized the agar surface, spawn was prepared by placing a piece of agar culture (1.5 × 2.0 cm) into sterilized (121 °C, 20 min) sorghum grains (Wageningen, the Netherlands). The spawn was incubated at 24 °C for 4 or 5 weeks. The chopped straw was soaked in water for 3 days at room temperature and excess water was drained off for 5 h. Adjustments were made based on the final moisture content of the straw – which ranged from 77 (MS1) to 80% (MS3) for each container (172 × 110 × 70 mm; Combiness, Nevele, Belgium) to contain 41.7 ± 0.3 g of dry matter. After autoclaving at 121 °C for 1 h, the straw was aseptically inoculated with the previously prepared spawn at 10% of the dry weight. Control (untreated) and fungal‐treated wheat straw were incubated in triplicate under solid‐state fermentation at 24 °C for 7 weeks in a climate‐controlled chamber (Wageningen University, the Netherlands). All weekly samples were freeze dried and ground over a 1 mm sieve using a cross beater mill (100AN: Peppink, Olst, Netherlands).

#### *Chemical analysis*

Each sample was analyzed for dry matter (DM; ISO 6496, 1999) and ash (ISO 5984, 2002) content. Crude protein was calculated by multiplying the nitrogen content (ISO 5983, 2005) by 6.25. The Van Soest *et al*.^19^ method was used to determine the cell‐wall composition. Neutral detergent fiber (NDF) was determined using a heat‐stable amylase (thermamyl) and alcalase; acid detergent fiber (ADF) and acid detergent lignin (ADL) were determined by boiling the sample in an acid detergent solution, and the latter was further treated with 72% v/v H_2_SO_4_. All fiber contents were corrected for ash. Hemicellulose was calculated as the difference between NDF and ADF, while cellulose was calculated as the difference between ADF and ADL. Reducing sugars in the ethanol‐extract of the straw were determined by measuring the oxidation reaction between the hydrolyzed monosaccharides with copper (II) and neocuproine at 460 nm. Absolute amounts (g) of each component were calculated from the remaining amount (g) of the freeze‐dried samples.

#### *Ergosterol estimation*

Fungal biomass was estimated by determining the content of ergosterol. Details of the procedure have previously been described.^5^, ^20^ Samples (∼200 mg) were saponified with 10% (1:9) KOH/methanol solution at 80 °C for 60 min. After cooling, the ergosterol was extracted from the samples through a series of mixing with 1 mL of water and 2 mL of hexane. The hexane layers from the same sample were collected by centrifuging and pooled into a tube. The pooled hexane layer was dried in a vacuum evaporation system (Rapidvap, Kansas, MO, USA) before re‐dissolving in methanol. The solution was filtered into a high‐performance liquid chromatography (HPLC) vial for Waters HPLC‐PDA analysis (Alliance HPLC system, Milford, USA). Cholecalciferol (vitamin D_3_) was used as an internal standard. The ergosterol peak was detected at 280 nm.

#### In vitro gas production (IVGP)

*In vitro* gas production (IVGP) was used to assess the ruminal degradability of the wheat straw. The gas production experiment was performed according to the procedures described by Cone *et al*.^21^ In brief, rumen fluid was collected from two non‐lactating cows that were fed 1 kg concentrate and grass silage *ad libitum*. The rumen fluid was filtered through a cheesecloth and mixed (1:2 v/v) with phosphate‐bicarbonate buffer solution, which also contained trace elements, hydrolyzed casein, redox indicator and reducing agent.^21^ The samples (0.5 g) were incubated in 60 mL of buffered rumen fluid for 72 h and the gas production was automatically registered. The gas‐production data were fitted to a biphasic model^22^ to determine the kinetic parameters (*A*_*n*_, *B*_*n*_, *C*_*n*_, *t*_*R*m*n*_, *R*_m*n*_), where *n* is the phase number (1 or 2). *A*_*n*_ is the asymptotic gas production (mL g^−1^ OM) of phase *n*; *B*_*n*_ is the half time of the maximum gas production (h); *C*_*n*_ is a parameter to determine the steepness of the curve; *t*_*R*m*n*_ is the time of the maximum fractional rate of substrate degradation (h), and *R*_m*n*_ is the maximum fractional rate of substrate degradation (h^−1^).

#### *Quantitative pyrolysis GC/MS with ^13^C lignin as internal standard*

Prior to Py‐GC/MS, ground wheat straw (1 mm) was ball‐milled in a MM200 mixer mill (Retsch, Haan, Germany) and biological triplicates were mixed to one replicate. Pyrolysis of the ball‐milled sample was carried out as previously described in detail by Van Erven *et al*.^17^ Briefly, 10 µL of a ^13^C lignin internal standard (IS) solution (1 mg mL^−1^) was mixed with ∼80 µg of sample and dried before analysis. Lignin‐derived pyrolysis products were monitored in full mass spectrometry mode on the two most abundant fragments per compound (both ^12^C and ^13^C). The area for each compound was normalized by dividing by the respective relative response factor (RRF). Relative response factor values were updated to system performance by recalculation to obtain an identical relative abundance of lignin‐derived pyrolysis products of the ^13^C IS added to a wheat straw reference sample. Lignin content (% w/w) was determined as the sum of lignin‐derived pyrolysis products, where RRF corrected areas for each compound were multiplied by the molecular weight of the respective compound and summed instead of the application of the published correction factor of 1.057.^17^ Relative abundances of lignin‐derived pyrolysis products were based on areas normalized for the ^13^C analogues from the IS present in the same sample to distinguish matrix and treatment effects. Areas were not corrected for molecular weight before relative abundance determination as previously described by Del Río *et al*.^23^ as RRF values are mole based. Compounds were classified according to their structural features (Table S1, File S1 in the supplementary material) and summed. All samples were prepared and analyzed in triplicate.

### Effect of different wheat cultivars

An independent experiment assessing the effects of different straw cultivars on the fungal pretreatment was carried out. Wheat straw from five winter wheat cultivars – *Britannia* (CV1), *Cellule* (CV2), *Henrik* (CV3), *Residence* (CV4) and *Tabasco* (CV5), were purchased from Limagrain (Rilland, Netherlands). Any remaining spike from the straw was removed prior to chopping. The wheat straw was chopped at 3 cm length. The chopped straw underwent the same processing and treatment procedures as previously described with the same fungal strains used as mentioned above. Solid state fermentation of the inoculated wheat straw was carried out at 24 °C for 7 weeks in a climate controlled room with minimum exposure to light. The weekly samples were weighed, freeze dried, and ground over a 1 mm sieve for further analysis. All samples were subjected to the same chemical and ergosterol analyses as well as to the IVGP procedure described above. No Py‐GC/MS was conducted on these samples.

### Statistics

Data from both experiments were independently analyzed by analysis of variance using the generalized linear model (GLM) in SAS 9.3, followed by post‐hoc multiple group comparisons using least significance differences. The statistical model for the ergosterol and IVGP data included the effect of levels in each factor (maturity stage 1 to 3; or cultivar 1 to 5), treatment (control, *C. subvermispora* and *L. edodes*), week, and the interaction effects of all three terms. For chemical analyses, the end point data were analyzed by excluding week and its interaction terms from the previous model. Analysis of pyrolysis data of different maturity stages reflected analytical rather than biological variances as three biological replicates of the same treatment (with an equal amount) were mixed prior to the analysis. The minimum significance threshold level was set at *P* < 0.05. Pearson's product–moment correlation (*r*) coefficients were also determined among the measured variables.

## RESULTS AND DISCUSSION

### Effect of different maturity stages

#### *Mass balances for the wheat straw at different maturities*

The dry matter, ash content, and the mass balances of different wheat straw maturities treated with CS and LE for 7 weeks are reported in Table 1. The water‐holding capacity of the straw was increased with straw maturity, as is evident by a 7% lower DM content of MS3 straw as compared to MS1 and MS2 (Table 1). However, the total amounts of OM were not different for the untreated straw at different maturities. To allow comparison across different maturity stages, the amounts of the quantified nutrients were expressed per 100 g of starting OM for the respective stages. For the untreated straws, higher amounts of ADL (12.2%) and cellulose (6%) were seen with increasing maturity. The untreated MS3 straw contained a significantly (*P* < 0.01) higher amount of ADL compared to the rest. Hemicellulose was significantly (*P* < 0.001) lower in the MS3 straw compared to its counterparts, whereas high (*P* < 0.01) amounts of free sugars and crude protein were observed for the MS1 straw.

**Table 1:** Dry matter, ash content and mass balances for wheat straw of different maturity stages, treated with *C. subvermispora* (CS) and *L. edodes* (LE) for 7 weeks

Maturity stage^a^	Treatment	DM (g kg^−1^)	Ash (g kg^−1^ DM)	Amount (g per 100 g of starting OM)^b^						
				OM	Cell	Hcell	ADL	Sugar	CP	NI
MS1	Control	184.3^a^	18.9^d^	100.0^a^	47.7^b^	34.4^a^	7.4^c^	0.7^d^	3.3^b^	6.6^d^
	CS	158.4^c^	24.7^ab^	78.7^c^	40.9^de^	8.3^e^	1.4^f^	1.8^bc^	3.5^a^	22.7^a^
	LE	156.9^c^	23.6^b^	74.7^d^	40.5^e^	11.6^c^	2.9^d^	1.8^bc^	3.2^bc^	14.5^c^
MS2	Control	183.3^a^	20.7^c^	100.0^a^	49.6^a^	34.1^a^	7.8^b^	0.5^e^	2.5^e^	5.5^d^
	CS	154.5^cd^	25.5^ab^	82.5^b^	42.3^d^	8.7^e^	1.4^f^	2.1^a^	3.1^c^	24.8^a^
	LE	150.7^d^	24.3^ab^	79.0^c^	44.4^c^	10.8^cd^	2.2^e^	1.9^b^	2.8^d^	16.9^b^
MS3	Control	171.3^b^	19.9^cd^	100.0^a^	50.7^a^	30.8^b^	8.4^a^	0.5^e^	2.6^e^	7.0^d^
	CS	150.9^d^	25.8^a^	82.2^b^	46.2^b^	6.4^f^	0.2^g^	2.1^a^	2.9^d^	24.5^a^
	LE	145.3^e^	25.3^a^	78.5^c^	46.8^b^	9.9^d^	1.7^f^	1.7^c^	2.8^d^	15.7^bc^
RMSE		2.36	0.96	0.74	0.89	0.67	0.23	0.06	0.09	1.27

a Maturity stages around harvest (MS1: Zadoks code 83; MS2: code 87; MS3: code 91).

b Calculated from the remaining materials for each treatment (g), using respective starting OM (week 0) for each maturity stage.

Values with different superscripts within a column are significantly (*P* < 0.05) different.

DM, dry matter; OM, organic matter; Cell, cellulose; Hcell, hemicellulose; ADL, acid detergent lignin; Sugar, free reducing sugar from ethanol‐extract of wheat straw; CP, crude protein (N × 6.25); NI, unaccounted OM; RMSE, root mean square error.

There were significant (*P* < 0.001) losses of OM after 7 weeks of fungal pretreatment (17.5% to 25.3%), with high losses observed for fungal‐treated MS1 straw. *Lentinula edodes* resulted in a significantly (*P* < 0.01) higher loss of OM than CS at any maturity stage. Overall, both fungi significantly (*P* < 0.001) degraded all cell‐wall components and increased the amount of soluble sugars. A lesser amount of cellulose was degraded when both fungi grew on MS3 as compared to MS1 (∼8.3% versus 14.6%, respectively). Interestingly, lignin degradation by both fungi was more pronounced on mature straw. The delignification by CS, for instance, was similar when grown on MS1 and MS2 straw (∼82%), whereas on MS3 straw, CS degraded 98.2% of the total amount of ADL. The figures for lignin degradation presented here were higher than those in previous reports (33% to 60%), which were carried out under similar treatment conditions,^5^, ^6^, ^7^ but at an unknown stage of maturity. Meanwhile, a similar trend was also observed for the hemicellulose with a higher percentage of degradation (66.2% to 79.3%) for both fungi. *Ceriporiopsis subvermispora* consistently resulted in higher level of degradation of ADL and hemicellulose than LE for all straw maturities. In addition, both fungi also increased (*P* < 0.001) the amount of free sugars in the treated straw, which may have arisen from the breakdown of the cell‐wall polysaccharides. The apparent susceptibility of a more mature straw to fungal delignification is of particular interest here. To investigate the delignification characteristics of both fungi, the samples from the control (week 0) and after 7 weeks of fungal pretreatment were subjected to quantitative pyrolysis coupled to gas chromatography and mass spectrometry, using ^13^C lignin as an internal standard ^13^C‐IS Py‐GC/MS analysis. There was an average variation of 8% in the ADL measurement of biological replicates of the same treatment. Hence, prior to Py‐GC/MS, the biological replicates were thoroughly mixed to allow analytical triplicates for detailed and more accurate lignin analysis.

#### *Quantitative ^13^C‐IS Py‐GC/MS of the wheat straw*

A total of 34 lignin‐derived compounds were released on pyrolysis and monitored (see supporting information, Table S1, File S1). The lignin content, as quantified using ^13^C‐IS Py‐GC/MS, and its structural features are summarized in Table 2. Overall, the untreated straws showed comparable amounts of lignin (23.8 g per 100 g OM), in contrast with the ADL method. It is inferred that mature straw may contain a high amount of recalcitrant residual lignin that was retained in the ADL fraction, which explains a higher ADL in MS3 straw than its counterparts. Acid detergent lignin is known to underestimate the total lignin content, as it does not take into account the acid‐soluble lignin.^24^ Both methods, however, seem in agreement on the extent of delignification by both fungi. *Ceriporiopsis subvermispora* showed a higher delignification capability on MS3 straw (87.6%) than MS1 (84.4%) and MS2 (82.4%). Similar observations were also recorded for LE‐treated MS3 straw (80.1%), as compared to MS1 and MS2 (∼75%).

**Table 2:** Characterization of lignin and its structural moieties in different straw maturities (MS), treated with *C. subvermispora* (CS) and *L. edodes* (LE) for 7 weeks, using quantitative ^13^C‐IS Py‐GC/MS

	MS1^a^			MS2			MS3		
Parameter	Control	CS	LE	Control	CS	LE	Control	CS	LE
Lignin content (% w/w DM)	23.7	4.7	8.1	22.6	4.8	6.9	23.5	3.5	5.9
Amount of lignin (g per 100 g OM)	24.2	3.8	6.2	23.1	4.1	5.6	24.0	3.0	4.8
Lignin aromatic unit (%)^b^									
H	8.9	13.3	11.1	9.3	13.3	11.9	8.8	13.9	11.4
G	62.6	61.3	63.3	62.5	59.3	62.9	61.2	59.3	62.4
S	28.5	25.5	25.6	28.1	27.5	25.2	30.0	26.8	26.2
S/G	0.46	0.42	0.40	0.45	0.46	0.40	0.49	0.45	0.42
Structural moieties (%)									
Unsubstituted	4.8	11.4	7.7	4.7	12.2	9.3	5.2	13.7	10.0
Methyl	2.0	3.7	2.9	2.1	3.8	3.0	2.3	4.5	3.5
Ethyl	0.1	0.2	0.2	0.1	0.2	0.2	0.2	0.2	0.2
Vinyl	29.9	24.8	28.4	30.5	23.9	27.3	30.4	20.3	26.9
C_*α*_‐oxidized	3.2	20.1	8.4	3.4	21.3	10.2	3.2	26.1	10.6
C_*β*_‐oxidized	1.6	3.7	2.3	1.5	3.7	2.6	1.6	3.9	2.7
C_*γ*_‐oxidized	57.0	46.8	52.0	56.2	46.8	51.1	55.7	47.8	49.8
Miscellaneous	1.9	1.9	2.1	1.9	1.8	1.8	2.0	1.7	2.0

a MS: maturity stages around harvest (MS1: Zadoks code 83; MS2: code 87; MS3: code 91).

b G: guaiacyl lignin subunit, H: *p*‐hydroxyphenyl unit, S: syringyl unit.

Values are averages of three technical replicates. No statistics were carried out on the data.

To explain this observation, we assessed the lignin structural features of all straws. The untreated MS3 straw contained 6% more syringyl (S) unit compounds than the other straws. This also resulted in a higher S to guaiacyl (G) ratio in MS3 (0.49) than MS1 and MS2 straws (∼0.45). Fungal pretreatment resulted in minor changes in lignin subunit composition, with slight preference of fungi towards attacking the S units, which is in agreement with previous reports.^25^, ^26^ The results also indicate that both fungi can degrade all lignin subunits at the magnitudes of lignin removal. The S‐units result in more linear structures and have a lower redox potential than the G‐ units,^23^, ^27^ which made them more susceptible to the fungal attack.^25^ Hence, the relatively high S/G ratio may partly explain the higher fungal delignification of MS3 straw. Nevertheless, the S/G ratio was poorly correlated with the total amount of lignin (Pearson's *r* = −0.17), due to a smaller change in the S/G ratio. Fungal pretreatment resulted in reduced amounts of vinyl products, mainly 4‐vinylguaicol (supplementary material, Table S1 in File S1). These compounds are also derived from ferulic acids that link lignin to its associated carbohydrates.^28^ The reduction of vinyl products was more pronounced on the more mature straw, particularly for the CS treatment. Other notable changes include the increase in unsubstituted and C_*α*_‐oxidized compounds with increasing straw maturity, particularly with the CS treatment, despite no difference being observed in the abundances of these compounds in the untreated straws. These observations clearly indicate more extensive degradation of inter‐unit linkages within the lignin macromolecule of the more mature straw, which resulted in residual lignin with low intact linkages.^25^, ^29^ The ADL and Py‐GC/MS assessments confirm the preference of both fungi for degrading the lignin of more mature straw – both by decreasing the amount of residual lignin and structural modifications.

#### *Fungal growth*

**Figure 1:**
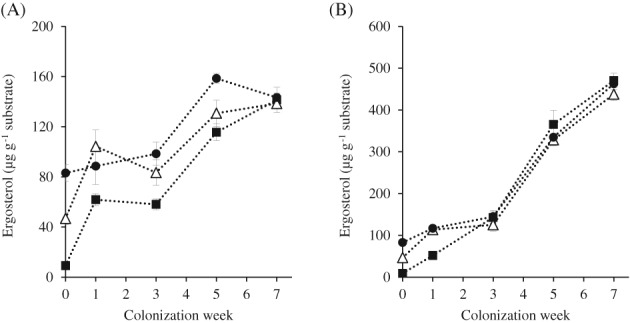
Growth (based on ergosterol data) of *C. subvermispora* (A) and *L. edodes* (B) on the wheat straw harvested at different maturity stages – MS1 (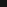), MS2 (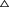) and MS3 (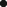), for 7 weeks. Error bars indicate standard deviations.

Successful colonization of the substrate is an important prerequisite for effective fungal pretreatment.^9^ We therefore assessed the growth of both fungi during the 7 week colonization period using an ergosterol assay (Fig. 1).^20^, ^30^ The baseline ergosterol content at week 0 was significantly (*P* < 0.001) lower in the untreated MS1 straw and increased with increasing maturity. The higher ergosterol content in MS3 straw may be due to its high water potential (high osmotic gradient), which leads to high activity and the growth of field fungi.^31^ Another possible explanation is post‐harvest fungal growth, which is a common occurrence.^32^ Although all processed straw was autoclaved prior to the fungal pretreatment, the persistence of ergosterol in the sterilized straw has been reported in several other studies.^5^, ^30^ The differences in the baseline ergosterol contents among the straw types produced unique colonization characteristics of both fungi, especially during the early growth stage. The growth of both fungi on MS3 at the beginning of the colonization weeks appeared slower, compared to their growth on MS1 and MS2. It is possible that existing ergosterol (or other components) belonging to the endogenous field fungi might have ‘masked’ the initial growth of CS and LE. On MS3 straw, both fungi might have recycled these endogenous compounds and incorporated them in their own biomass, erroneously indicating a slower growth or the inability of these fungi to colonize the straw during the early weeks. The variation in the fungal growth rate could be seen throughout the colonization period, especially for CS, which showed a low persistence in growth. This observation indicates a weaker colonization trait for this fungus on different straw types, as compared to LE.

#### *Assessing* in vitro *rumen degradability of the straw*

The effectiveness of both fungi in improving the rumen degradability of the straw was assessed by the IVGP technique, which was used as a decisive parameter in selecting the strains used in the present study.^16^ Table 3 summarizes the IVGP and its kinetic parameters for straw of different maturities, treated with the two fungal species. The changes in IVGP have been explained well by the changes in the cell‐wall composition of the substrate.^7^, ^33^ Although the control straws were noticeably different in their cell‐wall compositions, the IVGPs of all straw maturities were not different. There was no effect of maturity stage on the IVGP of the fungal‐treated wheat straws. *Ceriporiopsis subvermispora* and LE significantly (*P* < 0.05) improved the IVGP of all straw types by 33.6% and 20.4%, respectively, which was within the range of previous reports.^5^, ^16^ *Ceriporiopsis subvermispora* performed significantly (*P* < 0.05) better than LE in improving the degradability of the straw at all stages. The highest increase in the IVGP by both fungal pretreatments was observed on MS1 straw with 38.4% and 23.3% increases for CS and LE, respectively.

**Table 3:** *In vitro* gas production and its kinetic parameters for wheat straw of different maturity stages, treated with *C. subvermispora* (CS) and *L. edodes* (LE) for 7 weeks

Maturity stage^a^	Treatment	IVGP	Kinetic parameters					
			*A*_1_	*A*_2_	*B*_2_	*C*_2_	*t*_*R*m2_	*R*_m2_
MS1	Control	241.4^a^	32.0^a^	193.6^a^	13.93^c^	2.39^a^	15.99^bc^	0.087^a^
	CS	334.2^de^	61.4^b^	262.8^de^	10.52^a^	2.88^cde^	13.09^a^	0.143^c^
	LE	297.7^bc^	52.3^b^	231.5^b^	12.72^b^	2.74^b^	15.57^b^	0.112^b^
MS2	Control	250.6^a^	34.5^a^	198.1^a^	14.47^c^	2.50^a^	17.01^d^	0.088^a^
	CS	323.6^cde^	61.5^b^	253.2^cde^	10.70^a^	2.94^de^	13.39^a^	0.145^c^
	LE	307.0^bcd^	50.3^b^	243.8^bc^	13.15^b^	2.79^bc^	16.20^bcd^	0.111^b^
MS3	Control	254.5^a^	34.5^a^	202.3^a^	14.36^c^	2.48^a^	16.84^cd^	0.088^a^
	CS	339.5^e^	60.6^b^	269.8^e^	10.48^a^	3.00^e^	13.20^a^	0.152^c^
	LE	293.4^b^	37.3^a^	245.2^bcd^	13.06^b^	2.79^bc^	16.09^bc^	0.112^b^
RMSE		17.00	7.87	10.98	0.39	0.07	0.52	0.005

a Maturity stages around harvest (MS1: Zadoks code 83; MS2: code 87; MS3: code 91).

Values with different superscripts within a column are significantly (*P* < 0.05) different.

RMSE, Root mean square error; IVGP, cumulative *in vitro* gas production after 72 h (mL g^−1^ OM); *A*_1_, *A*_2_, asymptotic gas production (mL g^−1^ OM) phase 1 and 2, respectively; *B*_2_, half time to the maximum gas production of phase 2 (h); *C*_2_, parameters determine the curvature of the graph; *t*_*R*m2_, time of the maximum fractional rate of substrate degradation (h); *R*_m2_, maximum fractional rate of substrate degradation (h^−1^).

The kinetic parameters were determined using a biphasic model approach^22^ to differentiate the IVGP profile of all straws. This approach has been used to differentiate the fermentability of two fungal‐treated wheat straws with similar total IVGP.^5^ All fungus‐treated straws showed a better kinetic profile than the control – among others, a shorter half time to the asymptotic gas production (*B*_2_) and a steeper curve (greater *C*_2_). There was no effect of straw maturity on the kinetic parameters of the fungal‐treated straws, although a numerical increase of fractional fermentation rate at phase 2 (*R*_m2_) could be seen for CS‐treated straws with increasing maturity. This observation indicates that both fungi were very capable of improving the degradability of these straws to a similar extent.

### Effect of different cultivars

#### *Mass balances for wheat straw of different cultivars*

Table 4 summarizes the mass balances of the different wheat straw cultivars, treated and not treated with fungi. There was considerable variation in the amount of nutrients among the different straw cultivars, particularly in the DM and ash contents. The absolute amounts of OM in the starting materials were comparable among the different untreated straw cultivars (ranging from 36.3 to 37.6 g; Table 4). As in the previous experiment, the amount of nutrients was expressed per 100 g of starting OM. The starting amounts of all cell‐wall components were comparable among the different untreated straw cultivars, with CV5 high in lignin. Besides showing a high ash content, the untreated CV2 and CV3 straws contained lower amounts of CP than the other straw cultivars.

**Table 4:** Mass balances for wheat straw of different cultivars, treated with *C. subvermispora* (CS) and *L. edodes* (LE) for 7 weeks

Cultivar^a^	Treatment	DM (g kg^−1^)	Ash (g kg^−1^ DM)	Amount (g per 100 g of starting OM)^b^						
				OM	Cell	Hcell	ADL	Sugar	CP	NI
CV1	Control	173.9^ef^	26.9^k^	100.0^a^	51.1^abc^	32.9^a^	6.5^b^	0.5^h^	1.8^fg^	7.2^d^
	CS	156.9^gh^	35.0^hi^	87.5^e^	46.8^f^	9.6^g^	0.4^e^	2.4^a^	2.9^ab^	25.5^a^
	LE	154.2^h^	33.0^i^	88.0^e^	49.2^de^	12.2^f^	1.3^d^	2.3^ab^	2.8^ab^	20.3^bc^
CV2	Control	193.5^ab^	75.1^e^	100.0^a^	50.4^cde^	33.8^a^	6.3^b^	0.5^h^	1.5^gh^	7.5^d^
	CS	177.0^cdef^	85.1^c^	97.1^b^	49.7^cde^	19.9^b^	3.0^c^	1.8^e^	2.2^cdef^	20.6^bc^
	LE	186.5^bcde^	78.9^d^	96.2^b^	52.5^ab^	17.9^bcd^	3.3^c^	2.1^bcd^	2.1^def^	18.2^c^
CV3	Control	203.1^a^	84.7^c^	100.0^a^	50.9^bcd^	33.4^a^	6.1^b^	0.4^h^	1.2^h^	7.9^d^
	CS	189.0^abcd^	95.2^a^	96.6^b^	50.5^bcde^	19.3^bc^	2.8^c^	1.5^f^	2.2^cdef^	20.4^bc^
	LE	190.4^abc^	90.7^b^	95.7^bc^	52.5^ab^	17.4^cd^	3.3^c^	2.1^cd^	2.3^cde^	18.3^c^
CV4	Control	191.2^abc^	30.7^j^	100.0^a^	49.6^cde^	33.6^a^	6.6^ab^	0.7^g^	1.9^ef^	7.6^d^
	CS	163.2^fgh^	38.5^f^	96.4^b^	50.1^cde^	16.6^de^	2.9^c^	1.9^de^	2.6^bc^	22.2^b^
	LE	175.3^def^	35.8^gh^	94.2^cd^	52.8^a^	14.7^e^	2.8^c^	2.4^a^	2.4^cd^	19.1^c^
CV5	Control	192.2^ab^	29.9^j^	100.0^a^	49.2^de^	33.0^a^	7.2^a^	0.6^gh^	2.2^cde^	7.9^d^
	CS	170.1^fg^	37.3^fg^	95.4^bc^	48.3^ef^	16.0^de^	2.7^c^	2.0^de^	3.2^a^	23.1^ab^
	LE	171.0^fg^	35.2^ghi^	92.9^d^	50.7^bcd^	15.3^e^	2.9^c^	2.2^abc^	2.8^ab^	19.0^c^
RMSE		9.02	1.36	1.11	1.11	1.08	0.34	0.11	0.24	1.54

a CV1: *Britannia*; CV2: *Cellule*; CV3: *Henrik*; CV4: *Residence*; CV5: *Tabasco*.

b Calculated from the remaining materials for each treatment (g), using respective starting OM for each maturity stage.

Values with different superscripts within a column are significantly (*P* < 0.05) different.

DM, dry matter; OM, organic matter; Cell, cellulose; Hcell, hemicellulose; ADL, acid detergent lignin; Sugar, free reducing sugar from ethanol‐extract of wheat straw; CP, crude protein (N × 6.25); N.I, unaccounted OM. RMSE, root mean square error.

There were significant (*P* < 0.05) losses of OM in all fungal‐treated straws, with a relatively higher loss observed for CV1 straw (∼12.2%). The fungal degradation of lignin and hemicellulose for most straw cultivars was in the range of previous reports.^5^, ^6^, ^7^ The fungal‐treated CV1 straw, however, showed high losses of lignin (∼86.5%) and hemicellulose (∼67.0%). Previously, CS had been shown to be superior over LE in its delignification capability.^7^, ^16^ In the present study, the delignification capability of CS was surprisingly comparable to LE for most straw cultivars (56.2% versus 53.3%). *Ceriporiopsis subvermispora* degraded a significantly (*P* < 0.01) higher amount of lignin in CV1 than LE (93.5% versus 79.6%). Significant losses of cellulose were observed for both fungi for the CV1 straw. Other fungal‐treated straws showed slight changes in their cellulose contents but a significant increase in cellulose was observed for LE‐treated CV2 (4%) and CV4 straws (6.5%). Similar observations were also reported in several other studies.^6^, ^7^ The difficulties in the accurate quantification of cellulose with regards to the interference of fungal biomass have been described previously.^16^ Nonetheless, the variable observation in cellulose content among studies (based on the gravimetric method) triggers an intriguing question regarding how these different types and batches of straw affect the fungal growth and the extent of utilization of the polysaccharides.

#### *Fungal growth*

**Figure 2:**
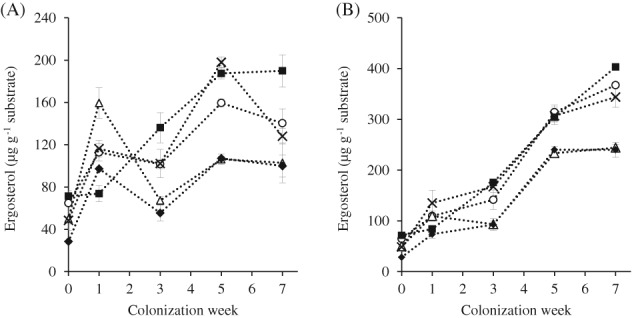
Growth (based on ergosterol data) of *C. subvermispora* (A) and *L. edodes* (B) on the wheat straw of different cultivars – CV1 (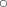), CV2 (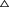), CV3 (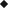), CV4 (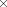) and CV5 (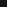) for 7 weeks. Error bars indicate standard deviations.

The growth of both fungi on wheat straw of different cultivars is illustrated in Fig. 2. The baseline ergosterol content varied considerably among all five cultivars, ranging from 28.4 µg g^−1^ (CV3) to 71.4 µg g^−1^ (CV5). *Lentinula edodes* showed a more consistent growth on all straw cultivars, as compared to CS, although its overall growth was noticeably lower on CV2 and CV3 straw. Similar to the growth on MS3 in an earlier experiment (with high baseline ergosterol content), the growth of both fungi were more challenged on the CV5 straw. Due to a characteristically smaller formation of the mycelium, CS was more affected by the variable baseline ergosterol content, as compared to LE. On CV2 and CV3 straws, the CS growth appeared ‘stunted’ after a rapid increase in ergosterol at week 1, before its growth continued at a slower rate towards the end of the colonization weeks. There were also significant (*P* < 0.05) decreases in the ergosterol content of CS grown on CV 1 and CV4 after week 5. These observations further indicate a weaker colonization trait of CS as compared to LE on various types of straw used.

#### *Assessing* in vitro *rumen degradability of the straw*

The IVGP and its kinetic parameters for straw of different cultivars treated with CS and LE are summarized in Table 5. There were no differences in the IVGP between the different untreated straws, although a significantly (*P* < 0.05) lower *R*_m2_ was observed for CV3 straw as compared to CV1 and CV5. Although both fungi significantly (*P* < 0.001) increased the IVGP of the straws (as compared to controls), the magnitude of the effects was significantly (*P* < 0.001) affected by the different straw cultivars. The IVGP of the fungus‐treated CV2 and CV3 was noticeably lower than the other treated straw cultivars with the difference between CS‐treated CV3 and CV4 being significant (*P* < 0.05). The *R*_m2_ of CV2 and CV3 treated with both fungi were also significantly (*P* < 0.05) lower than the other treated straw cultivars. Overall, CS treatment resulted in a significantly (*P* < 0.05) higher increase in the IVGP than LE (∼43 versus 29%).

**Table 5:** *In vitro* gas production and its kinetic parameters for wheat straw of different cultivars, treated with *C. subvermispora* (CS) and *L. edodes* (LE) for 7 weeks

Cultivar^a^	Treatment	IVGP	Kinetic parameters					
			*A*_1_	*A*_2_	*B*_2_	*C*_2_	*t*_*R*m2_	*R*_m2_
CV1	Control	223.8^a^	16.9^b^	199.9^a^	14.45^de^	2.41^a^	16.68^bc^	0.085^bcd^
	CS	308.4^fg^	49.4^g^	255.6^ef^	11.20^a^	2.86^d^	13.91^a^	0.135^j^
	LE	282.7^bcde^	32.1^de^	243.7^de^	12.57^b^	2.67^bcd^	15.22^ab^	0.110^h^
CV2	Control	205.4^a^	6.0^a^	191.3^a^	15.38^ef^	2.41^a^	17.76^cde^	0.080^abc^
	CS	294.6^defg^	41.9^efg^	245.0^de^	13.31^bcd^	2.55^abc^	15.79^bc^	0.099^efg^
	LE	269.4^bc^	33.5^de^	224.6^bc^	14.39^de^	2.53^abc^	17.01^bc^	0.090^de^
CV3	Control	203.9^a^	9.2^ab^	183.5^a^	16.96^g^	2.44^a^	19.68^e^	0.073^a^
	CS	289.2^cdef^	36.7^def^	240.7^cde^	14.14^cde^	2.51^abc^	16.63^bc^	0.091^def^
	LE	260.7^b^	27.5^cd^	219.6^b^	14.81^e^	2.49^abc^	17.37^cd^	0.086^cd^
								
CV4	Control	213.1^a^	9.4^ab^	192.8^a^	16.28^fg^	2.47^ab^	19.04^de^	0.077^ab^
	CS	314.4^g^	37.9^def^	266.9^f^	12.86^bc^	2.73^cd^	15.69^abc^	0.111^h^
	LE	281.0^bcde^	30.4^d^	241.1^cde^	13.40^bcd^	2.59^abc^	16.04^bc^	0.099^fg^
CV5	Control	209.9^a^	18.2^bc^	183.1^a^	15.12^ef^	2.44^ab^	17.56^cd^	0.082^bcd^
	CS	305.2^efg^	47.4^fg^	252.9^ef^	12.23^ab^	2.89^d^	15.24^ab^	0.124^i^
	LE	269.6^bcd^	34.9^de^	225.8^bc^	12.58^b^	2.62^abc^	15.12^ab^	0.107^gh^
RMSE		13.38	6.00	10.28	0.71	0.13	1.07	0.005

a CV1: *Britannia*; CV2: *Cellule*; CV3: *Henrik*; CV4: *Residence*; CV5: *Tabasco*.

Values with different superscripts within a column are significantly (*P* < 0.05) different.

RMSE, Root mean square error; IVGP, cumulative *in vitro* gas production at 72 h (mL g^−1^ OM); *A*_1_, *A*_2_, asymptotic gas production (mL g^−1^ OM) phase 1 and 2, respectively; *B*_2_, half time of the maximum gas production of phase 2 (h); *C*_2_, parameters determine the curvature of the graph; *t*_*R*m2_, time of the maximum fractional rate of substrate degradation (h); *R*_m2_, maximum fractional rate of substrate degradation (h^−1^).

Correlating the IVGP with the fungal growth (ergosterol) is rather complex, although its relationship was statistically significant (*r* = 0.56; *P* < 0.001). *Ceriporiopsis subvermispora,* which possessed a weaker colonization trait on any straw type, resulted in a more degradable straw than LE. Hence, the growth ‘inconsistency’ seen in CS more likely suggests a higher dynamic nutrient utilization and recycling of the field fungi biomass, contributing to an apparently slow growth rate. It does not indicate the inability of this particular fungus to colonize the different straw types successfully. When scaling‐up the bioprocess in practice, the slow growth of CS may lead to a problem with undesirable microbial growth on the substrate. Nevertheless, an improved and optimized inoculation method can be used to ensure a quick colonization of the substrate. In both trials above, these high‐potential strains showed a high persistency in improving the degradability of the straw, although different straw cultivars affected the IVGP of the CS and LE treatments. Significant (*P* < 0.05) correlations were found between the IVGP and the cell‐wall compositions in both trials, with stronger correlations to lignin (*r* ∼ −0.91) and hemicellulose (*r* ∼ −0.92) than to cellulose (*r* ∼ 0.51). Nonetheless, the current results indicate that a careful assessment has to be made when describing the chemical changes (using the unspecific gravimetric method) and relate them to the subsequent changes in IVGP. As mentioned above, the interference of fungal biomass has to be taken into account. Based on this consideration, the IVGP is, therefore, the most potent method for assessing the success and effectiveness of a particular fungus in improving the ruminal degradability of different straw types.

## CONCLUSION

Different straw types influenced the characteristics of *C. subvermispora* and *L. edodes* in degrading lignin. A more pronounced degradation of lignin was observed on mature straw, which was further confirmed by quantitative ^13^C‐IS Py‐GC/MS of the wheat straw. Variable results were observed for lignin degradation with different straw cultivars. Both high‐potential strains of *C. subvermispora* and *L. edodes* were able to improve the ruminal degradability of wheat straw, regardless of the various straw types (maturity and cultivar) investigated. The magnitude of the effect, however, was only affected by different straw cultivars but not by different maturity stages when harvested. Under all circumstances, *C. subvermispora* was more capable than *L. edodes* of improving the degradability of wheat straw. *Lentinula edodes* was more adapted for colonizing different straw types than *C. subvermispora*.

## Supporting information

File S1.
