# Supplementary material for: Improving ruminal digestibility of various wheat straw types by white‐rot fungi
Source: J Sci Food Agric. 2018 Oct 16;99(2):957–65. doi: 10.1002/jsfa.9320 (PMC6587845; doi:10.1002/jsfa.9320)
Supplement: Supplementary file 1 — File S1. [file JSFA-99-957-s001.docx]

Supporting information for:

**Improving ruminal digestibility of various wheat straw types by white-rot fungi**

Nazri Nayan^a*^, Gijs van Erven^b^, Mirjam A. Kabel^b^, Anton S.M. Sonnenberg^c^, Wouter H. Hendriks^a^ and John W. Cone^a^

^a^Animal Nutrition Group, Wageningen University & Research, De Elst 1, 6708 WD, Wageningen, The Netherlands

^b^Laboratory of Food Chemistry, Wageningen University & Research, Bornse Weilanden 9, 6708 WG, Wageningen, The Netherlands

^c^Plant Breeding, Wageningen University & Research, Droevendaalsesteeg 1, 6708 PB, Wageningen, The Netherlands

*Correspondence: nazri.nayan@live.com. Animal Nutrition Group, Wageningen University & Research. P.O. Box 338, 6700 AH, Wageningen, the Netherlands

| **Table S1.** Identities and relative abundances of lignin-derived compounds in different straw maturities (MS), treated with *C. subvermispora* (CS) and *L. edodes* (LE) for 7 weeks | | | | | | | | | | | | | | | | |
| --- | --- | --- | --- | --- | --- | --- | --- | --- | --- | --- | --- | --- | --- | --- | --- | --- |
| Label | Compound | Origin | Ret. Time | MW ^12^C | Structure* | MS1 | | |  | MS2 | | |  | MS3 | | |
|  |  |  |  |  |  | Control | CS | LE |  | Control | CS | LE |  | Control | CS | LE |
|  |  |  |  |  |  |  |  |  |  |  |  |  |  |  |  |  |
| 1 | Phenol | H | 10 | 94 | Unsub | 0.7 | 3.4 | 1.9 |  | 0.8 | 3.5 | 2.5 |  | 0.7 | 4.4 | 2.5 |
| 2 | Guaiacol | G | 10.29 | 124 | Unsub | 2.2 | 5.2 | 3.4 |  | 2.2 | 5.6 | 4.2 |  | 2.4 | 6.2 | 4.6 |
| 3 | 2-Methylphenol | H | 11.25 | 108 | Methyl | 0.2 | 0.7 | 0.5 |  | 0.2 | 0.9 | 0.6 |  | 0.2 | 1.0 | 0.7 |
| 4 | 4-Methylphenol | H | 12.19 | 108 | Methyl | 0.4 | 1.3 | 0.8 |  | 0.4 | 1.3 | 1.0 |  | 0.4 | 1.8 | 1.0 |
| 5 | 4-Methylguaiacol | G | 12.97 | 138 | Methyl | 0.8 | 1.0 | 1.0 |  | 0.8 | 0.9 | 0.9 |  | 0.9 | 1.0 | 1.0 |
| 6 | 2,4-Dimethylphenol | H | 13.41 | 122 | Methyl | 0.1 | 0.2 | 0.1 |  | 0.1 | 0.3 | 0.2 |  | 0.1 | 0.4 | 0.2 |
| 7 | 4-Ethylguaiacol | G | 15.14 | 152 | Ethyl | 0.1 | 0.2 | 0.2 |  | 0.1 | 0.2 | 0.2 |  | 0.2 | 0.2 | 0.2 |
| 8 | 4-Vinylguaiacol | G/FA | 16.59 | 150 | Vinyl | 19.7 | 15.7 | 18.3 |  | 19.8 | 15.1 | 17.5 |  | 20.0 | 12.8 | 17.7 |
| 9 | 4-Vinylphenol | H/PCA | 16.7 | 120 | Vinyl | 7.3 | 7.1 | 7.5 |  | 7.8 | 6.8 | 7.4 |  | 7.3 | 5.9 | 6.7 |
| 10 | Eugenol | G | 17.2 | 164 | Misc | 0.2 | 0.2 | 0.2 |  | 0.2 | 0.2 | 0.2 |  | 0.2 | 0.2 | 0.2 |
| 11 | Syringol | S | 17.94 | 154 | Unsub | 1.9 | 2.8 | 2.3 |  | 1.8 | 3.1 | 2.6 |  | 2.1 | 3.1 | 2.9 |
| 12 | *cis*-Isoeugenol | G | 18.57 | 164 | Misc | 0.1 | 0.2 | 0.1 |  | 0.1 | 0.1 | 0.1 |  | 0.1 | 0.2 | 0.1 |
| 13 | *trans*-Isoeugenol | G | 19.83 | 164 | Misc | 0.8 | 0.9 | 1.0 |  | 0.8 | 0.8 | 0.8 |  | 0.8 | 0.8 | 0.9 |
| 14 | 4-Methylsyringol | S | 20.18 | 168 | Methyl | 0.6 | 0.5 | 0.5 |  | 0.6 | 0.4 | 0.4 |  | 0.7 | 0.4 | 0.5 |
| 15 | Vanillin | G | 20.27 | 152 | C*_α_* -ox | 1.0 | 2.9 | 1.6 |  | 1.0 | 2.8 | 1.7 |  | 0.9 | 3.0 | 1.7 |
| 16 | Homovanillin | G | 21.73 | 166 | C*_β_* -ox | 0.5 | 1.0 | 0.7 |  | 0.5 | 1.0 | 0.7 |  | 0.5 | 1.0 | 0.7 |
| 17 | Acetovanillone | G | 22.19 | 166 | C*_α_* -ox | 0.2 | 1.4 | 0.7 |  | 0.3 | 1.4 | 0.8 |  | 0.2 | 1.7 | 0.9 |
| 18 | 4-Hydroxybenzaldehyde | H | 22.92 | 122 | C*_α_* -ox | 0.1 | 0.5 | 0.3 |  | 0.1 | 0.5 | 0.3 |  | 0.1 | 0.5 | 0.2 |
| 19 | 4-Vinylsyringol | S | 23.24 | 180 | Vinyl | 2.9 | 1.9 | 2.6 |  | 2.9 | 2.0 | 2.4 |  | 3.1 | 1.7 | 2.5 |
| 20 | Guaiacylacetone | G | 23.41 | 180 | C*_β_* -ox | 0.3 | 1.1 | 0.6 |  | 0.3 | 1.1 | 0.7 |  | 0.3 | 1.1 | 0.7 |
| 21 | Guaiacyl vinyl ketone | G | 24.36 | 178 | C*_α_* -ox | 0.1 | 0.3 | 0.2 |  | 0.1 | 0.3 | 0.2 |  | 0.1 | 0.4 | 0.2 |
| 22 | Vanilloyl acetaldehyde | G | 24.6 | 194 | C*_α_* -ox,  C*_γ_* -ox | 0.2 | 7.7 | 2.2 |  | 0.2 | 7.9 | 3.3 |  | 0.3 | 10.8 | 3.2 |
|  |  |  |  |  |  |  |  |  |  |  |  |  |  |  |  |  |

| **Table S1.** (*continued*) | | | | | | | | | | | | | | | | |
| --- | --- | --- | --- | --- | --- | --- | --- | --- | --- | --- | --- | --- | --- | --- | --- | --- |
|  |  |  |  |  |  |  |  |  |  |  |  |  |  |  |  |  |
| 23 | *trans*-4-Propenylsyringol | S | 26.08 | 194 | Misc | 0.9 | 0.6 | 0.8 |  | 0.8 | 0.6 | 0.7 |  | 1.0 | 0.5 | 0.7 |
| 24 | Syringaldehyde | S | 26.65 | 182 | C*_α_* -ox | 0.7 | 1.1 | 0.9 |  | 0.8 | 1.2 | 0.8 |  | 0.8 | 1.2 | 0.9 |
| 25 | *cis*-Coniferyl alcohol | G | 26.72 | 180 | C*_γ_* -ox | 1.2 | 0.9 | 1.2 |  | 1.1 | 0.8 | 1.1 |  | 1.2 | 0.7 | 1.0 |
| 26 | Homosyringaldehyde | S | 27.64 | 196 | C*_α_* -ox | 0.3 | 0.5 | 0.4 |  | 0.3 | 0.5 | 0.4 |  | 0.3 | 0.6 | 0.4 |
| 27 | Acetosyringone | S | 28.09 | 196 | C*_α_* -ox | 0.6 | 1.2 | 0.9 |  | 0.6 | 1.3 | 0.9 |  | 0.6 | 1.3 | 1.0 |
| 28 | *trans*-Coniferyl alcohol | G | 28.43 | 180 | C*_γ_* -ox | 33.1 | 20.6 | 29.9 |  | 32.9 | 19.0 | 28.3 |  | 31.3 | 17.1 | 26.7 |
| 29 | *trans*-Coniferaldehyde | G | 28.8 | 178 | C*_γ_* -ox | 2.0 | 2.1 | 2.2 |  | 2.1 | 2.0 | 2.3 |  | 2.0 | 2.2 | 2.5 |
| 30 | Syringylacetone | S | 29.02 | 210 | C*_α_* -ox | 0.5 | 1.2 | 0.7 |  | 0.4 | 1.2 | 0.7 |  | 0.5 | 1.2 | 0.8 |
| 31 | Syringoyl acetaldehyde | S | 29.78 | 224 | C*_α_* -ox,  C*_γ_* -ox | 0.2 | 5.0 | 1.7 |  | 0.3 | 5.9 | 2.1 |  | 0.2 | 7.3 | 2.5 |
| 32 | *cis*-Sinapyl alcohol | S | 31.95 | 210 | C*_γ_* -ox | 1.1 | 0.6 | 0.8 |  | 1.0 | 1.0 | 0.8 |  | 1.1 | 0.8 | 0.9 |
| 33 | *trans*-Sinapyl alcohol | S | 33.66 | 210 | C*_γ_* -ox | 17.2 | 7.9 | 11.9 |  | 16.9 | 7.7 | 10.8 |  | 17.8 | 6.9 | 10.7 |
| 34 | *trans*-Sinapaldehyde | S | 33.88 | 208 | C*_γ_* -ox | 1.9 | 2.1 | 2.1 |  | 1.7 | 2.6 | 2.4 |  | 1.8 | 2.0 | 2.4 |
|  |  |  |  |  |  |  |  |  |  |  |  |  |  |  |  |  |
| G: guaiacyl lignin unit, H: p-hydroxyphenyl unit, S: syringyl unit, FA: ferulic acid, PCA: *p*-coumaric acid; Ret. Time: Retention time; MW ^12^C: Molecular weight  * Unsub: unsubstituted; Methyl, ethyl, vinyl: substituted compounds; Misc: miscellaneous structures; C*_α_* -ox, C*_α_* -ox, C*_α_* -ox: compounds with oxidation at C*_α_*, C*_β_* and C*_γ_* position, respectively. | | | | | | | | | | | | | | | | |
